# Barriers, interventions, and recommendations: Improving the genetic testing landscape

**DOI:** 10.3389/fdgth.2022.961128

**Published:** 2022-11-01

**Authors:** E. J. Dusic, Tesla Theoryn, Catharine Wang, Elizabeth M. Swisher, Deborah J. Bowen, EDGE Study Team

**Affiliations:** ^1^ Institute of Public Health Genetics, Department of Biostatistics, University of Washington, Seattle, WA, United States; ^2^ Department of Community Health Sciences, Boston University School of Public Health, Boston, MA, United States; ^3^ Department of Obstetrics and Gynecology, Fred Hutchinson Cancer Center, University of Washington, Seattle, WA, United States; ^4^ Department of Bioethics, University of Washington, Seattle, WA, United States; ^5^ Beth Devine, Department of Pharmacy, University of Washington, Seattle, WA, United States; ^6^ Barbara Norquist, Department of Obstetrics & Gynecology, University of Washington Medical Center, University of Washington, Seattle, WA, United States; ^7^ Brian Shirts, Department of Laboratory Medicine & Pathology, University of Washington Medical Center, University of Washington, Seattle, WA, United States; ^8^ Mariebeth Velasquez, Department of Family Medicine, University of Washington Medical Center, University of Washington, Seattle, WA, United States; ^9^ Michael Raff, Genomics Institute, MultiCare Health System, Tacoma, WA, United States; ^10^ Jeannine M. Brant, Clinical Science & Innovation, Billings Clinic, Billings, MT, United States

**Keywords:** cancer, genetics, primary care, population testing, genetic testing

## Abstract

Individual, provider, clinic, and societal level barriers have been shown to undermine the potential impact of genetic testing. The current approach in the primary care setting places an exorbitant burden on both providers and patients. Current literature provides insight into how to address barriers across multiple levels (patient, provider, clinic, system) and at multiple stages in the testing process (identification, referral, counseling, and testing) but interventions have had limited success. After outlining the current approach to genetic testing in the primary care setting, including the barriers that prevent genetic testing uptake and the methods proposed to address these issues, we recommend integrating genetic testing into routine medical care through population-based testing. Success in efforts to increase the uptake of genetic testing will not occur without significant changes to the way genetic services are delivered. These changes will not be instantaneous but are critical in moving this field forward to realize the potential for cancer risk genetic assessment to reduce cancer burden.

## Introduction

There is a pressing need to better integrate genetic testing for hereditary cancer risk into primary care settings. Genetic testing is the future of preventative medicine, but currently remains an untapped resource for patients who may benefit from additional cancer surveillance or risk-reduction. The clinical impact of this testing depends on the effective identification of interested at-risk patients, successful delivery of testing, and follow-up care for individuals with positive results.

**Figure 1:**
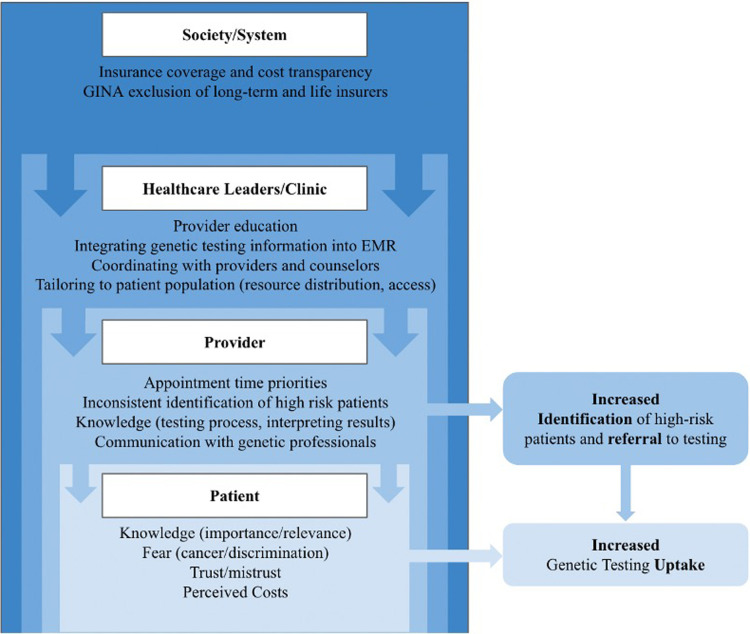
Multilevel barriers to accessing genetic testing for monogenic, hereditary cancer risk.

Individual interventions have had varying degrees of success in identifying strategies that are capable of increasing hereditary risk assessment and genetic testing uptake (1, 2). However, with existing barriers on the patient, provider, and systems level, targeted interventions have had limited efficacy. As such, we argue that until genetic testing for cancer is integrated as a routine part of medical care at a population level, interventions will continue to be limited in their capacity to increase genetic testing uptake and subsequent follow-up care. The successful integration of routine assessment for hereditary cancer risk into an existing healthcare setting will require input from, and action by, multiple stakeholders (Figure 1).

The purpose of this paper is to provide an overview of the current model of genetic testing in the primary care setting, describe multilevel barriers to genetic testing, and assess proposed interventions from the existing body of literature. We conclude with suggestions for a future action plan that promotes population-based genetic testing and informs practical decisions about how to integrate genetic testing for cancer into the primary care setting.

## Current primary care model of genetic testing for cancer risk

The current primary care model relies on a patient-provider interaction to start the testing process, an interaction that often occurs by chance. A patient may fill out a family history or the patient may ask a direct question about their risk that cues the provider to ask about family history and other risk factors. Sometimes, a patient-provider general discussion reveals clues that may lead an astute provider to pursue a cancer-risk conversation (3, 4). Any of these interactions would shape the provider's decision about appropriate testing and can lead to a subsequent referral to a genetics expert or to directly order a test, depending on the clinic's resources.

If a provider does determine that a patient would benefit from genetic testing, they can either directly order a test or refer the patient to a genetics professional. Even if they choose referral, extensive patient education may be needed regarding purpose, benefit, and risk of genetic testing to motivate the patient to complete the genetics consultation. If the provider orders the test directly, they are responsible for choosing the correct test, answering any patient concerns, including those surrounding privacy, cost, and explaining implications of positive and negative results (5–7). When results are ready, the test requisitioner must explain their implications, and then the primary care provider is responsible for integrating the genetic risk assessment into follow-up care. This places an unreasonable amount of responsibility on primary care providers who have minimal genetics training and have many competing priorities, leaving behind a large proportion of patients with a strong family history of hereditary cancer who would benefit from genetic testing (8, 9).

## Barriers presented by the current model

Barriers, as it applies to genetic screening and testing, refers to the individual, provider, system, and policy level hurdles an individual must overcome to access genetic testing and navigate the process of genetic testing. Investigators have done extensive work identifying the multilevel barriers within the current model of genetic testing for cancer risk (Figure 1). Shen and colleagues (10) conducted a systematic review of the barriers to population genetic screening as a whole, while other studies have evaluated challenges specific to genetic testing for cancer (3, 6, 11–15).

Individual-level barriers include the perceived cost of genetic testing, knowledge and beliefs about the genetic testing process, fear related to being at an increased risk for cancer, fear of genetic discrimination, and distrust in the medical system (16–18). Additionally, patients may have confusion over different testing options (such as lab tests vs. direct-to-consumer genetic testing) (19). These challenges make it difficult for patients to ascertain definitive answers related to medical genetic testing, leading to reduced genetic testing uptake (17, 18).

At the level of the provider, primary care providers (PCPs) play an important role in helping patients overcome individual-level barriers. However, to do so they need to prioritize conversations about cancer risk during appointments, consistently identify high-risk patients, clarify how to obtain and order genetic testing, learn the nuances of result interpretation, improve communication with the clinical genetics services, and establish how to utilize test results to inform patient care (5, 6, 13, 14, 20–22). This highlights another issue – the need for extensive physician education on risk assessment and genetics, including continuing medical education. Even with improved educational opportunities, it seems unrealistic to expect primary care providers to gain that level of comfort with cancer genetics when it is only offered on a case-by-case basis.

An additional barrier is that many PCPs do not have a thorough understanding of the role and availability of genetics professionals to effectively collaborate with them or connect patients to these resources (13, 21). Furthermore, the current shortage of genetic counselors compounds the challenge, particularly for providers in rural areas. This lack of communication with or access to genetics professionals, along with providers' inability to consistently determine which patients are high-risk, contributes to an overall under-referral and under-utilization of genetic testing; an issue that is exacerbated in historically marginalized populations who have lower referral and genetic testing rates from their providers (14).

At the systems level, genetic testing processes have not been integrated fully into EHR systems. The nuanced nature of genetic testing information, referral recommendations, coordination of communication between providers and specialists, and post-test care pathways for patients identified with hereditary risk all pose challenges to the production of an effective EHR (23). Alongside these issues are challenges involving various test types and inconsistent nomenclatures from different labs.

Even if an individual patient overcomes these multilevel barriers, they may still face policy-level barriers related to lack of insurance coverage and the cost of genetic testing. The cost of genetic testing services is not well understood by patients or providers. Although the cost has decreased dramatically in recent history, out-of-pocket costs of genetic testing can still range anywhere from $100 to $2,000 depending on the type of test that is ordered and how much is covered by insurance (24). Additionally, it is unclear to patients and providers whether insurance will cover the cost of subsequent genetic counseling services and follow up care, which can expose patients to additional unexpected costs. Medicare and Medicaid have long been criticized for underinsuring preventative care. Medicare does not cover the cost of pre-diagnosis genetic testing for hereditary cancer risk, excluding one test for colorectal cancer, Cologuard™ (25). Research has shown that many patients who could benefit from genetic testing for hereditary cancers are being missed because Medicare will not cover the cost of pre-symptomatic genetic testing until after the cancer is diagnosed (15, 26).

Additionally, concerns about discrimination by insurers make individuals hesitant to undergo genetic testing (27). While the Genetic Information Nondiscrimination Act (GINA) prevents discrimination by health insurance companies based on genetic test results, it does not apply to long term care insurance, disability insurance, or life insurance (28).

## Solutions from existing literature

All these factors are intricately intertwined and contribute to a low uptake rate of genetic testing for hereditary cancer, particularly amongst cancer-free patients. Failure to address these issues across multiple levels has contributed to an inability to reduce cancer mortality rates (12). A comprehensive, population-based, and standardized model that considers and balances challenges at each level and can be adapted to unique healthcare systems is critical to overcoming this problem on a large scale. Studies to date have failed to address multilevel barriers, target different aspects of the genetic testing process, and have largely been conducted in urban, high-resourced facilities under optimal conditions.

Still, this is a prolific field of study with many suggested solutions existing at each level. Bednar and colleagues (2) conducted a comprehensive review of tested interventions over the past 20 years (2000–2020) and found over 60 interventions targeting different barriers throughout the testing process. Among the 16 interventions aimed at increasing genetic testing uptake, only 5 showed positive results (28–32), while several studies demonstrated high rates of genetic testing but with no supporting statistical analysis (33–35). Additionally, only 5 of the 16 studies recruited participants who had not previously received a cancer diagnosis (28, 34, 36–38), while only 2 of these 5 were aimed at the general public or a general clinic population (28, 38). The first was conducted by the Centers for Disease Control and Prevention who conducted a direct-to-consumer mass marketing campaign aimed to increase uptake in genetic testing for BRCA1 and BRCA2 variants (28). Although they did not report genetic testing rates, they report that there was a statistically significant increase in genetic testing uptake in target cities. The second, conducted by DeFrancesco et al., implemented family history screening methods based on NCCN guidelines in community-based OB/GYN clinics (38). Patients who were eligible were then offered genetic counseling and genetic testing. Although no statistical tests were performed, genetic testing uptake increased from 0.5% pre-implementation to 4.0% post-implementation.

While ideally patients will be captured prior to a cancer diagnosis, this has not always been the case under our current model of genetic testing. As such, interventions have been tested to address this issue, also with varying success. For example, Uyar et al. (31) recruited patients with ovarian cancer from an academic medical center to refer them to genetic counseling and genetic testing. This intervention included provider education, EHR integration, patient education and navigation, and tumor board documentation. After performing tests of significance, they found that genetic testing rates significantly increased from 27% pre-intervention to 82% post-intervention. We can certainly use this and other types of studies as a model for how to improve genetic testing uptake for all patients.

Effective aspects of all 16 interventions included direct patient engagement through mass marketing or online testing information, integration of a genetic counselor or health navigator into the genetic testing process, and physician education and support. Importantly, an aspect of physician support that will be critical as we move towards a population-based screening model is effective integration of genetic information in the EHR (39–43). Additionally, telemedicine and telephone delivery strategies (expanded since the onset of the COVID-19 pandemic) offer the opportunity to expand accessibility of genetic counseling services (44, 45), although reported success of these types of interventions varies (2) and concerns about equity in access remain (46).

Still, there are remaining limitations, including: a lack of follow-up time with patients, lack of detail in risk assessment strategies, largely White and high-income study populations (and other generalizability issues), and privacy concerns (39–41, 44, 45, 47, 48). None of these interventions have addressed larger systems-level issues such as insurance coverage and policies that protect against discrimination by long-term care insurance or life insurance.

To build on previous efforts, we have implemented the EDGE Study (NCT04746794) which is a multilevel intervention with input from varied stakeholders, to improve population-based screening methods for hereditary cancer (49). All active participants identified in participating clinics are given the opportunity to take an online familial risk assessment survey and are offered genetic testing if they are eligible. Components of this intervention include in clinic and online screening tools, free genetic testing, telephone genetic counseling, and clinical care pathways developed for patients who receive positive genetic test results. Results of this intervention are pending, but the main goal is to eliminate the primary care provider as the gatekeeper of genetic testing, drawing them into the clinical paradigm to implement well-defined care plans for patients identified with pathogenic variants that increase cancer risk.

Genetic testing, generally, has been historically inaccessible to underrepresented and underserved populations (6, 50, 51), particularly for individuals of low socioeconomic status and/or low health literacy. The genetic tests offered by the EDGE Study were free, which addresses individual-level barriers (perceived cost of genetic testing) and systems-level barriers (lack of insurance coverage). However, this is not a real-world model in current practice, and the costs associated with genetic testing will have to be addressed with larger societal interventions.

During the EDGE study, individuals are offered the opportunity to discuss their concerns or questions regarding genetic testing with research or clinic staff (49). PCPs do not have to prioritize the time in their visit because the familial risk assessment survey is taken online or while patients are waiting for their appointment to begin. The use of this online risk assessment is easily integrated into EHR and ensures consistent screening methods for all patients, as shown in other studies (9, 39). As a part of the EDGE Study, physicians participated in an educational course, earning continuing medical education credits, during which they learned more about the process of genetic testing and when genetic testing would be appropriate for their patients. If a patient receives a positive test result, the provider is given a gene-specific care pathway that outlines recommended cancer surveillance and/or prevention measures. EDGE has also created a protocol for healthcare systems to scan genetic test results into EHR as soon as they are released to providers. This type of tool can be implemented in diverse clinic environments to improve the communication of testing results.

## Population-based testing as the future of preventative medicine

While these interventions have been successful in increasing genetic testing uptake, their success has ultimately been limited by the multilevel barriers prolific in our current testing model. Offering genetic testing on a case-by-case basis, as we do currently, relies on the ability of healthcare providers to identify eligible patients with fidelity and move them through the process with ease. Meeting these conditions has proven untenable in many healthcare systems, even with certain interventions. As such, we must consider shifting to a population-based model in which all patients are offered genetic testing for hereditary cancer risk as part of their routine medical care.

A population-based testing model will address some testing barriers by design and others through systematization. Currently, providers fail to identify patients who meet eligibility criteria, and the eligibility criteria fail to capture all individuals who would benefit from genetic testing (52). These barriers are made obsolete through population-based testing. Many remaining barriers can be addressed through effective systematization using the lessons learned from existing interventions. Routinization of the genetic testing process will destigmatize genetic testing. Routine testing should increase providers' ability to order and interpret genetic test results, allow clinics to establish clear workflows, and improve patients' understanding of genetic testing. However, this will rely on investment in infrastructure, integration with EHR systems, hiring of appropriate personnel, and extensive education programs (53) – all shown to be effective through previous intervention efforts.

Studies evaluating population-based models have found that population-based testing identifies carriers that would be missed by traditional criteria, does not result in increased psychosocial concerns, can result in clinically actionable information, and can be cost-effective if implemented at 30 years old or earlier (52, 54–56). In population testing, genetic counseling is generally reserved for the post-test setting for those identified with positive results (i.e., pathogenic variants), similar to providing specialized counseling for an individual with high cholesterol on routine screening.

Until the scaffolding exists to establish population-based testing, streamlined genetic testing for patients with certain cancers (ie breast, ovarian, and cancers under 50) must remain a priority for interventions. Additionally, it is possible that individuals opt out of genetic testing as a preventative tool but still need a route to testing should they develop cancer. As such, there must be an effective clinical care pathway to receive streamlined genetic testing for those with a personal history of cancer. Hopefully, as the fear and stigma associated with genetic testing decreases and population-based testing expands, the need for streamlined testing will become less significant.

Many cancer prevention or surveillance tools are used in routine medical care (pap smears, low-dose CT scans, hepatitis C screenings, colonoscopies, mammograms) but genetic testing for cancer risk has not been utilized in the same way, thus limiting our capacity to prevent and detect hereditary cancer. Genetic indicators of risk should not be treated as though they are significantly different from other risk indicators and genetic testing should be integrated into primary care accordingly.

## Discussion

The joint guidelines put forth by the American College of Medical Genetics (ACMG) and the National Society of Genetic Counselors (NSGC), the President's Cancer Panel, and the extensive work in intervention research highlight the urgency of increasing genetic testing uptake rates for individuals who are eligible for, and interested in, genetic testing (57, 58). There is also strong empirical evidence synthesized in National Comprehensive Cancer Network (NCCN) screening guidelines, which highlight specific genes that could be included for genetic testing (Table 1) (59, 60). As cancer continues to burden individuals in the U.S., effective and creative ways to holistically address the multilevel barriers that prevent people from accessing genetic testing are crucial.

**Table 1:** Synthesis of genes and associated cancers.

Group Name	Gene	Main Cancers
Breast and Ovarian Cancer	BRCA1	breast, ovarian, prostate, and pancreatic
	BRCA2	breast, ovarian, prostate, and pancreatic
	PALB2	breast, ovarian, and pancreatic
	ATM	breast, ovarian, prostate, and pancreatic
	NBN	breast and ovarian
	BRIP1	breast and ovarian
	RAD51C	breast and ovarian
	RAD51D	breast and ovarian
Li Fraumeni	TP53	breast, colon, central nervous system, bone, and soft tissue
Breast and Gastric	CDH1	breast and gastric
Colon (Lynch)	MLH1	colon, uterine, ovarian, pancreatic, urothelial
	MSH2	colon, uterine, ovarian, pancreatic, urothelial
	MSH6	colon, uterine, ovarian, pancreatic, urothelial
	PMS2	colon and uterine
	EPCAM	colon, uterine, ovarian, pancreatic, urothelial
Cowden Syndrome	PTEN	breast, uterine, thyroid, endometrial, kidney, and melanoma
Breast and Colon	CHEK2	breast and colon
Breast	BARD1	breast
	NF1	Breast and peripheral nerve
Colon	APC	colorectal
Colon (Juvenile Polypsis Syndrome)	BMPR1A	colon and stomach
	SMAD4	colon and stomach
Colon (Adenomatous Polypsis)	GREM1	colon
	POLD1	colon
	POLE	colon
Colon (MUTYH-Associated Polypsis)	MUTYH	colon
Colon (Peutz-Jeghers Syndrome)	STK11	breast, ovarian, pancreatic, colon, stomach, small intestine, cervix, uterus, testes, and lung
Pancreatic	CDKN2A	pancreas and melanoma

The current model in the primary care setting is ineffective and fails to provide testing to eligible, interested patients. It places the burden of identifying high-risk patients on the provider or on the motivated patient, ignoring larger systemic and societal barriers an individual must overcome to access genetic testing and exacerbating existing healthcare disparities. Primary care providers, in our current medical landscape, are not given the tools and information to facilitate genetic testing for their patients. An existing body of work offers solutions to address multilevel barriers. However, there are limitations and testing uptake remains low. As demonstrated in previous work and by the methodology of the EDGE Study, future interventions must simultaneously address these barriers at as many levels as possible. Most of these barriers could be eliminated through a systematic population-based model.

Larger systemic and societal barriers, as well as the interaction between these levels, will take more time and effort to address, with input necessary from multiple stakeholders. Future interventions should target the challenges in expanding to population-based genetic testing and can start from the ground up - working within the system to address patient, provider, and clinic barriers while pushing for systemic- and policy-level change.

## EDGE Study Team

Beth Devine, Barbara Norquist, Brian Shirts, Mariebeth Velasquez, Michael Raff, Jeannine M. Brant.
